# Update on rotavirus vaccine underperformance in low- to middle-income countries and next-generation vaccines

**DOI:** 10.1080/21645515.2020.1844525

**Published:** 2020-12-17

**Authors:** Benjamin Lee

**Affiliations:** Vaccine Testing Center and Translational Global Infectious Diseases Research Center, University of Vermont College of Medicine, Burlington, VT, USA

**Keywords:** Rotavirus, rotavirus vaccines, oral vaccine, vaccine performance, low- to middle-income countries, developing countries

## Abstract

In the decade since oral rotavirus vaccines (ORV) were recommended by the World Health Organization for universal inclusion in all national immunization programs, significant yet incomplete progress has been made toward reducing the burden of rotavirus in low- to middle-income countries (LMIC). ORVs continue to demonstrate effectiveness and impact in LMIC, yet numerous factors hinder optimal performance and evaluation of these vaccines. This review will provide an update on ORV performance in LMIC, the increasing body of literature regarding factors that affect ORV response, and the status of newer and next-generation rotavirus vaccines as of early 2020. Fully closing the gap in rotavirus prevention between LMIC and high-income countries will likely require a multifaceted approach accounting for biological and methodological challenges and evaluation and roll-out of newer and next-generation vaccines.

## Introduction

Rotaviruses are a leading cause of pediatric diarrheal disease and mortality worldwide, with rotavirus-associated deaths in children <5 years old estimated to be 128,500–215,000 yearly.^1-5^ Oral rotavirus vaccines (ORV) have demonstrated remarkable efficacy and effectiveness in high-income countries, but they suffer from reduced performance in low- to middle-income countries (LMIC). In this review, we will provide an LMIC-specific update on recent estimates of the performance of currently approved ORVs, review factors affecting ORV performance, and discuss the status of newer and next-generation vaccines. This review will not discuss cost-effectiveness or vaccine supply and delivery issues, which are nicely summarized elsewhere.^6^

## Background

Rotaviruses are non-enveloped, double-stranded RNA viruses belonging to the *Reoviridae* family. Intact virions are composed of a complex, triple-layered icosahedral capsid and contain a genome of 11 segments encoding 12 viral proteins.^7^ Rotaviruses have traditionally been classified using a binary nomenclature based on the two outer capsid structural proteins: the surface glycoprotein VP7 (G) forms the shell of the outer capsid layer, out of which protrude multiple copies of VP4 (P), a protease-sensitive spike that is responsible for attachment to cellular receptors.^8^

Rotavirus infection causes acute gastroenteritis (AGE), characterized by vomiting, watery diarrhea, and often fever, leading to dehydration which may be fatal in severe cases without timely rehydration.^9^ The major targets of infection are the mature enterocytes of the small intestinal villi, although enteroendocrine cells may also be infected.^10,11^ Pathogenesis involves both osmotic and secretory diarrhea via malabsorption by infected enterocytes, alteration of intracellular calcium homeostasis leading to disruption of cytoskeleton and tight junction integrity, an enterotoxin-like effect modulated by non-structural protein 4 (NSP4), and stimulation of the enteric nervous system.^12^ Transmission is presumed to be mainly fecal-oral, although spread by contaminated water and fomites are also likely.^10,13^ In the pre-vaccine era, virtually all humans were infected during early childhood, with subsequent natural immunity developing after several rounds of childhood infection; for unclear reasons, children in lower-income settings appear to require a greater number of infections to gain less complete immunity.^14,15^

In high-income countries, AGE due to rotavirus infection was historically one of the most significant causes of pediatric morbidity and health-care utilization.^16^ Effective disease control in the United States, for example, was not achieved until the late 2000s after ORV introduction,^17^ indicating that good hygiene and sanitation alone are insufficient to control transmission. Thus, improvements in community sanitation and public health infrastructures in LMIC are unlikely to substantially decrease rotavirus incidence, compounding the challenge of diarrheal disease control in regions where ORVs underperform. These settings shoulder a disproportionate burden of global rotavirus-associated mortality, with only four countries (India, Nigeria, Pakistan, Democratic Republic of Congo) suffering nearly half of all rotavirus deaths.^5^

## Current oral rotavirus vaccines

Four ORVs are currently pre-qualified by the World Health Organization (WHO) for global use: RotaTeq (Merck & Co., Inc., Kenilworth, NJ), Rotarix (GlaxoSmithKline Biologicals, Rixensart, Belgium), Rotavac (Bharat Biotech International Ltd., Hyderabad, India), and Rotasiil (Serum Institute of India Pvt. Ltd., Pune, India). All are oral, live-attenuated vaccines; selected characteristics of each are presented in Table 1. Both RotaTeq and Rotarix demonstrated excellent vaccine efficacy in clinical trials conducted in higher-income settings,^19,20^ but markedly reduced vaccine efficacy was observed in clinical trials in LMIC.^21–24^ Despite reduced efficacy, in high-incidence settings vaccination still provides a significant overall reduction in severe disease, prompting the WHO to universally recommend ORV inclusion in all national immunization programs in 2009.^25^

**Table 1.** Overview of oral, live-attenuated rotavirus vaccines pre-qualified by the World Health Organization as of Jan 2020

Vaccine	Manufacturer	Composition	Dosage	Formulation	Storage^a^	EPI schedule	WHO pre-qualification^a^
RotaTeq	Merck & Co., Inc.	Pentavalent human-bovine mono-reassortants: human G1, G2, G3, G4, P[8] on bovine rotavirus (WC3) backbone	2–2.8 x 10^6^ IU/serotype	Liquid	2–8 C, 24 mo	6, 10, 14 weeks	Oct 2008
Rotarix	GlaxoSmithKline Biologicals	Monovalent G1P[8] (strain RIX4414) human rotavirus	≥10^6^ CCID_50_	Liquid	2–8 C, 24 mo	6, 10 weeks	Mar 2009
Rotavac^b^	Bharat Biotech International Ltd.	Monovalent G9P[11] (strain 116E) natural human-bovine reassortant	≥10^5^ FFU	Liquid	−20 C, 60 mo After thaw: 2–8 C, 6 mo	6, 10, 14 weeks	Jan 2018
Rotasiil^c^	Serum Institute of India Pvt. Ltd.	Pentavalent human-bovine mono-reassortants (BRV-PV): human G1, G2, G3, G4, G9 on bovine rotavirus (UK) backbone	≥10^5^^.^^6^ FFU/serotype	Lyophilized	2–8 C, 30 mo	6, 10, 14 weeks	Sep 2018
Rotasiil Thermo	Same	Same	Same	Lyophilized	25 C, 30 mo	Same	Jan 2020

Abbreviations: CCID_50_, cell culture infective dose 50; EPI, Expanded Programme on Immunizations; IU, international units; FFU, focus-forming units; WHO, World Health Organization

a. Ref^18^

b. Rotavac 5D: storage at 2–8 C but not yet pre-qualified by WHO

c. Rotasiil: liquid formulation requiring storage at 2–8 C not yet pre-qualified by WHO

### Rotarix and RotaTeq

Following introduction of Rotarix and RotaTeq, real-world vaccine effectiveness studies continued to support the observation that these vaccines provide substantial benefit yet underperform in LMIC compared to high-income settings. In a comprehensive systematic review and meta-analysis using data published prior to December 2, 2016, Jonesteller et al. reported a median vaccine effectiveness for Rotarix of 57% in countries with high child mortality, which are typically LMIC, vs 84% in low child mortality countries; for RotaTeq, median vaccine effectiveness was 45% and 90% in high child mortality and low child mortality countries, respectively.^26^ Since then, additional studies of Rotarix or RotaTeq effectiveness in LMIC report generally similar findings, with point estimates for adjusted effectiveness against rotavirus-associated AGE in children <5 ranging up to 68% (Table 2).^27–35^

**Table 2.** Summary of recent oral rotavirus vaccine effectiveness studies in LMIC

				Adjusted vaccine effectiveness (95% CI)		
Country	Vaccine	Setting	Age (years)	Any rotavirus gastroenteritis	Severe rotavirus gastroenteritis	Ref
Bangladesh	Rotarix	Hospital, Outpatient	<2	41% (23–55)	43% (22–58)	^27^
Burkino Faso	RotaTeq	Hospital	<5 6–11	35% (−15-63) 58% (10–81)	39% (−18-68) NR	^28^
Lebanon	Rotarix, RotaTeq	Hospital	<5	68% (50–80)	NR	^29^
Malawi	Rotarix	Hospital	<5	62% (28–80)	75% (41–89)	^30^
			<12 mo	75% (45–89)	83% (54–94)	
Philippines	Rotarix	Hospital	<5	62% (26–80)	67% (18–87)	^31^
Tanzania	Rotarix	Hospital	<2	49% (−30-80)	66% (−2-89)	^32^
Tanzania	Rotarix	Hospital	<5	75% (−8-94)	NR	^33^
				85% (27–97)^a^	NR	
Thailand	Rotarix	Hospital	<18 mo	88% (76–94)^b^	NR	^34^
Zimbabwe	Rotarix	Hospital, ED	1–5	−48% (−148-11)	−38% (−164-28)	^35^
			6–11 mo	61% (21–81)	68% (13–88)	

Rotavirus detection by stool rotavirus enzyme immunoassay except as otherwise noted.

Abbreviations: NR, not reported

^a^Detection by qPCR

^b^Detection by polyacrylamide gel electrophoresis

Despite decreased effectiveness in LMIC, substantial reductions in rotavirus-associated and all-cause AGE hospitalizations were observed in virtually all settings following national-level introduction of Rotarix or RotaTeq. Recently, Burnett et al. analyzed manuscripts of vaccine impact published prior to December 31, 2019 and found that median reductions in rotavirus hospitalizations or emergency department (ED) visits and all-cause AGE hospitalization in children <5 years in high child-mortality countries were 50% (IQR, 41–65) and 26% (IQR, 20–50).^36^ The largest study utilized the Global Rotavirus Surveillance Network (GRSN), which was established by WHO in 2008 with funding from Gavi, the Vaccine Alliance. Data from 305,789 cases in children <5 years admitted to the hospital with AGE from 2008 to 2016 were analyzed, representing 198 sites in 69 countries from all six major WHO regions (African Region, Region of the Americas, South-East Asia Region, European Region, Eastern Mediterranean Region, and Western Pacific Region). Overall, the study found a 39.6% (95% CI 35.4–43.8) relative reduction in the proportion of children admitted for AGE due to rotavirus post-vaccine introduction compared to pre-vaccine introduction.^37^ In Latin America, data from sentinel sites in Bolivia, El Salvador, Guatemala, Honduras, Paraguay, and Venezuela showed a mean reduction of 16% (95% CI 10–22) in the proportion of acute diarrhea samples positive for rotavirus.^38^ In Africa, percent reduction in rotavirus hospitalizations or emergency department visits was 46% among countries that had introduced ORV by 2013, and was 34% in countries that had introduced it later.^39^

Decreases in rotavirus-associated or all-cause AGE mortality in children following vaccine introduction have also been reported. In Malawi, a 31% reduction (95% CI, 1–52) was observed in all-cause AGE mortality following Rotarix introduction,^40^ and in Bolivia, a 53% reduction (95% CI, 47–56) in the proportion of deaths due to all-cause AGE was observed.^41^ In the previously mentioned meta-analysis by Burnett et al., median reduction in AGE mortality was 37% (IQR, 24–41) for children <5 years in high child-mortality countries.^36^ An estimated 134,714 (IQR, 112,321–154,654) hospitalizations and 20,986 (IQR, 18,924–22,822) deaths were prevented in 2016 in the 29 African countries that had introduced rotavirus vaccine by 2014; if all African countries had introduced vaccine, the numbers of hospitalizations and deaths prevented could have both been over twice these estimates.^42^ In Asia, ORVs introduction to all 43 Asian countries is estimated to decrease yearly rotavirus-associated hospitalizations by 710,000 (49% decline) and rotavirus-associated deaths by 35,000 (40% decline).^43^

Indirect vaccine effects among unvaccinated individuals, such as reductions in rotavirus incidence among unvaccinated individuals (e.g. herd immunity) have also been noted in many high-income settings, but are infrequently observed in LMIC.^44^ However, there may be additional indirect effects of vaccine introduction that may be unmeasured in typical impact studies. For example, in Bangladesh, which has a high burden of AGE hospitalizations and a chronic shortage of hospital beds, ORV use was estimated to free over 600 hospital beds per year, improving access to care for critical non-diarrheal pediatric illnesses.^45^

### Rotavac and Rotasiil

More recently, two ORVs manufactured in India, Rotavac, and Rotasiil, received WHO pre-qualification in 2018, after both demonstrated efficacy in clinical trials conducted in India (Rotavac, Rotasiil) and Niger (Rotasiil). A randomized, double-blind, placebo-controlled Rotavac trial performed from 2011 to 2012 in three sites in India demonstrated vaccine efficacy of 53.6% (95% CI, 35.0–66.9) against severe rotavirus gastroenteritis in the first year of life and 55.1% (39.9–66.4) until 2 years of age.^46,47^ A second formulation called Rotavac 5D has been developed and released, offering a ready-to-use liquid formulation stored at 2–8 C (versus Rotavac, which must be stored frozen for long-term storage), although it is not yet WHO pre-qualified.

Rotasiil is a lyophilized, heat-stable ORV that has been shown to be stable when stored up to 18 months at 37 C-40 C,^48^ and was tested in two separate trials. A randomized, double-blind, placebo-controlled trial was performed among infants in Niger from 2014 to 2015; vaccine efficacy against severe rotavirus gastroenteritis was 66.7% (95% CI, 49.9–77.9).^49^ A similar trial conducted at six sites in India from 2014 to 2015 reported vaccine efficacy against severe rotavirus gastroenteritis of 36% (95% CI, 11.7–53.6).^50^ Rotasiil has demonstrated lot-to-lot consistency with similar immunogenicity to that of Rotarix, as assessed by serum rotavirus-specific IgA (RV-IgA) antibody concentration.^51^ A ready-to-use liquid formulation has been developed which demonstrated immunogenicity (RV-IgA) non-inferiority to lyophilized Rotasiil with lot-to-lot consistency, but must be maintained at 2–8 C.^52^ This formulation is not yet WHO pre-qualified. The discrepancy between the efficacy estimates for the Niger study and the India study are unclear. Both studies were designed and powered for a primary endpoint of vaccine efficacy after a pre-determined target number of cases was achieved, and different protocols for vaccine storage temperatures were used in each study but these seem unlikely to be contributing factors.^49,50^

India has introduced Rotavac and Rotasiil at the national level, with roll-out having occurred in stages by state.^53^ Outside of India, as of early 2020 Palestine^54^ and Benin had introduced Rotavac and Democratic Republic of Congo had introduced Rotasiil, with a number of additional countries reportedly planning introduction or transition – Rotavac or Rotavac 5D: Egypt, Nigeria, Ghana, Sao Tome; Rotasiil, lyophilized: Mali, Uzbekistan; Rotasiil, liquid: Burkina Faso (Carl Kirkwood, personal communication).

### Locally approved vaccines: Rotavin and Lanzhou lamb rotavirus (LLR) vaccine

Two countries, China and Vietnam, have developed vaccines approved and licensed for national use. In China, LLR (Lanzhou Institute of Biological Products, Lanzhou, China), a G10P[15] strain, is recommended once annually in children from 2 months to 3 years of age. It has been available in China since 2000, but lack of inclusion in China’s universal vaccine program and lack of formal phase 3 efficacy trials hamper understanding of the vaccine’s true performance.^55^ Single-dose effectiveness has been estimated from 34.9% (95% CI, 5.3–55.3) to 73.3% (95% CI, 61.2–81.6) in children <5 years, with improved performance observed with earlier vaccination.^56–60^ In Vietnam, Rotavin M-1, (POLYVAC, Hanoi, Vietnam), an attenuated G1[P8] strain of human rotavirus, has been licensed since 2012, based solely on immunogenicity data.^61^

Updated formulations of these vaccines are currently undergoing or have recently completed evaluation. A phase 3 trial to compare Rotavin-M1, which is stored frozen, with a newer liquid formulation simply called Rotavin, is reportedly in progress (ClinicalTrials.gov NCT0370336). Very recently, LLR3 (Lanzhou Institute of Biological Products, Lanzhou, China), a new G2, G3, G4 trivalent human-lamb mono-reassortant vaccine based on Lanzhou lamb rotavirus vaccine, was evaluated in a phase 3 clinical trial in China. Vaccine efficacy against any rotavirus AGE was 56.6% (95% CI, 47.5–60.1) and 70.3% (95% CI, 60.6–77.6) against severe rotavirus AGE through two epidemic seasons following vaccination.^62^ These estimates are similar to vaccine efficacies measured in phase 3 clinical trials of Rotarix in China,^63^ and slightly lower than findings for RotaTeq.^64^

## Factors affecting oral vaccine performance

A number of factors have been proposed to contribute to poor ORV performance among children in LMIC, such as maternal antibodies, micronutrient deficiencies, gut dysbiosis, coinfections, environmental enteric dysfunction, and genetic factors.^65,66^ Most studies assessing factors related to ORV performance have utilized immunologic endpoints, typically RV-IgA seroconversion or concentration. RV-IgA is a suboptimal correlate of protection for ORVs in LMIC,^67–71^ so the effects of many factors on clinical protection remain incompletely understood. With this limitation in context, targeted interventions to improve ORV response have generally been unsuccessful (as further discussed below). Only two interventions, delay of the first ORV dose and separation of the first ORV dose from the first dose of oral polio vaccine have consistently demonstrated improvements in RV-IgA concentration or seroconversion.^72^ Unfortunately, these interventions would require significant adjustment to the WHO Expanded Programme on Immunizations (EPI) schedule, limiting feasibility.

### Vaccine schedules and dosing

#### Delayed dosing

The EPI schedule recommends ORV vaccination at 6 and 10 weeks (Rotarix) or 6, 10, and 14 weeks of age (RotaTeq, Rotavac, Rotasiil). The rationale for delayed dosing is that it may mitigate the inhibitory effects of maternal antibodies, as further discussed below. In systematic reviews and meta-analyses, delayed dosing of Rotarix, defined as the first vaccine dose administered at or beyond 10 weeks of age, was associated with improved RV-IgA seroconversion.^72,73^ Limited data also suggest improved clinical efficacy of delayed dosing. In Bangladesh, a delayed dosing schedule of 10 and 17 weeks demonstrated higher than expected efficacy against severe rotavirus diarrhea of 73.5% (95% CI, 45.8–87.0), but this was not directly compared to standard EPI scheduling.^22,74^ Pooled analyses from multiple Rotarix and RotaTeq studies suggested very modest improvements in protection from severe rotavirus gastroenteritis in children receiving a delayed first dose or increased interval between doses.^75^ Whether delayed dosing can truly improve vaccine efficacy may require additional dedicated, adequately powered clinical trials.

#### Additional doses

A related issue is the potential benefit of additional vaccine doses. Assessments of an additional dose or doses of Rotarix at 14 weeks of life or later on ORV immunogenicity have yielded inconsistent conclusions. In Ghana, increased RV-IgA seroconversion and geometric mean concentration (GMC) was seen among infants who received an additional dose of Rotarix at 14 weeks compared to infants who received Rotarix at 6 and 10 weeks only.^76^ However, a similar study in Pakistan was unable to demonstrate any effect, while a study in India found no differences with five doses of Rotarix compared to three.^77,78^ These studies suggest that the utility of this strategy may be location-specific. A study conducted at two sites in Africa (South Africa and Malawi) did demonstrate a slight improvement (not statistically significant) in year one vaccine efficacy with three doses compared to two doses among infants at the South Africa site, but not among infants in Malawi.^79^ The initial WHO recommendation for a two-dose Rotarix regimen included review of these year one efficacy data from these two sites.^25^ Subsequently, improved vaccine efficacy through 2 years of age was noted at both study sites in the three-dose group, as well as slightly increased RV-IgA seroconversion rates and GMC.^23,79,80^ However, these data alone were felt to be insufficient to recommend universal adoption of a three-dose regimen, although there was a call for additional research on the topic.^81^

#### Booster doses

Vaccine efficacy appears to be lower during the second year of life among children in LMIC,^82,83^ prompting investigations of a booster dose of ORV to counter waning immunity. A third (booster) dose of Rotarix administered with measles-mumps-rubella vaccine at 9 months was evaluated in Bangladesh. No effects on measles or rubella antibody titers were observed, and significant increases were seen in RV-IgA and RV-IgG seropositivity rates and geometric mean titer (GMT) in Rotarix booster recipients.^84^ A similar study in Mali was conducted to administer a booster dose of RotaTeq with yellow fever, measles, and meningococcal A vaccines at 9 months. Seroresponses to measles and meningococcal A vaccine were similar in RotaTeq booster recipients compared to control, and RotaTeq recipients had significant increases in RV-IgA and RV-IgG concentration and seroresponse rates; the study was unable to demonstrate non-inferiority to yellow fever seroresponse (as defined by ≥4-fold increase in titer), but responses to yellow fever vaccine were inexplicably quite low in both groups, making the clinical significance of this result unclear. These findings suggest potential for extending the duration of vaccine protection, but the overall effect may be fairly modest: mathematical modeling suggests that a 9- or 12-month ORV booster might avert 4,000–19,600 deaths (3–16% reduction) among children aged 1–2 per year globally.^85^

#### Size of vaccine inoculum

The potential effect of increasing vaccine inoculum has been widely discussed but few studies have directly addressed this topic in LMIC. For Rotarix, the effect of increasing vaccine inoculum, ranging from 1 × 10^4^^.^^1^ to 1 × 10^6^.^4^ focus-forming units (FFU)/dose, on seroconversion and RV-IgA GMC reached a plateau in studies from Europe, North America, Singapore, and Latin America.^86–89^ Early RotaTeq studies demonstrated a dose–response effect (range, 2.41 × 10^6^ to 2.69 × 10^7^ plaque-forming units/dose) of vaccine inoculum on G1-specific neutralizing antibody titers among children in Finland, although similar rates of RV-IgA seroconversion were seen.^90^^2^xrefSimilarly, increased seroconversion was observed at higher dosages for both Rotavac (1x10^5^ vs 1 × 10^4^ FFU/dose) and Rotasiil (range, 1 × 10^5^ to 1 × 10^6.^^4^ FFU/dose) in early studies in India.^91,92^ Our group recently performed a randomized-controlled trial comparing double the standard dose of Rotarix to standard dosing (1x10^6.^^3^ vs 1 × 10^6^ FFU/dose) among infants in Dhaka, Bangladesh. No differences were observed in RV-IgA seroconversion in high- versus standard-dose recipients (46% and 42%, respectively) or in GMC (30.4 units/mL vs 22.8 units/mL, respectively).^93^ The overall increase in vaccine inoculum was modest, which may help explain the apparent lack of benefit.

### Maternal antibodies

#### Maternally-derived serum RV-IgG

The most plausible explanation for the beneficial effect of delayed ORV dosing noted above is the inhibitory effect of maternally derived, transplacentally acquired serum antibodies (RV-IgG). Higher levels of RV-IgG or neutralizing antibodies at the time of vaccination have consistently and convincingly been associated with decreased ORV immunogenicity.^69,76,77,94–96^ Delayed dosing may provide additional time for waning of maternal antibodies, diminishing their effect on infant ORV response. However, the apparent benefits of delayed dosing would need to be weighed against the logistical challenges associated with alterations to the EPI schedule, along with the potential increase in early episodes of rotavirus AGE in regions with significant incidence at very young ages.^97,98^

#### Breast milk antibodies and antiviral factors

The role of breast milk on ORV response is less clear. Both breast milk antibodies and non-antibody breast milk components, such as lactoferrin, lactadherin, and human milk oligosaccharides (HMOs) diminish the infectivity of rotaviruses, including vaccine strains, *in vitro*.^99–101^ Formula-fed infants in Mexico achieved higher RV-IgA concentrations compared to breast-fed infants, even though RV-IgA geometric mean titer (GMT) in breast-fed infants was still quite robust.^102^ The hypothesis that withholding breastfeeding at the time of vaccination could potentially improve infant vaccine response was tested in multiple LMICs. In clinical trials in India and South Africa, no differences in vaccine immunogenicity could be detected between infants in whom breastmilk was withheld around the time of vaccination compared to those breastfed at the time of vaccination,^103,104^ and in a similar study from Pakistan, RV-IgA seroconversion was paradoxically increased in the immediate breastfeeding arm.^105^ Given the clear health benefits of breastfeeding and the lack of effect from short-term withholding of breastfeeding, further studies targeting breastfeeding are difficult to justify.

### Histoblood group antigens (HBGA)

HBGA status, particularly secretor status, clearly affects susceptibility to rotavirus gastroenteritis. Secretor status is determined by the *FUT2* gene, which encodes an α-[1,2]-fucosyltransferase that controls the expression of various 2-fucosylated HBGAs on mucosal surfaces (such as the gut) and in exocrine secretions (including saliva and breast milk). In the gut, these HBGAs are proposed molecular receptors for viral attachment. Non-secretors, who do not express these fucosylated targets, have been found to be far less susceptible to AGE due to P[8] and P[4] rotaviruses, and individuals who lack Lewis (*FUT3*)-derived antigens are more susceptible to P[6] viruses.^106–110^ Therefore, a recent area of scrutiny has been the role of infant secretor status on ORV response, particularly for Rotarix, an attenuated P[8] strain. Studies from Ghana and Pakistan have reported decreased rates of RV-IgA seroconversion among infant secretors,^111,112^ while no such differences were found in studies from Bangladesh and Malawi.^113,114^ Additional findings from the Bangladesh study offer a potential explanation. In this study, infants born to maternal non-secretors had significantly increased risk of seroconversion compared to infants born to maternal secretors, and the greatest affect was observed in infants who were themselves secretor-positive but born to non-secretor mothers, with 73% seroconversion in secretor-positive infants born to genotype-confirmed non-secretor mothers, compared to 23% in those born to genotype-confirmed secretor mothers.^114^ A proposed mechanism for this effect is that *FUT2*-dependent HMOs present in breast milk may act as decoy receptors for vaccine-strain virus; this could explain why vaccinated infants without interference from breast milk ligands but who expressed 2-fucosylated HBGAs on the gut surface (i.e. secretor infants born to maternal non-secretors) had the highest rates of vaccine seroconversion.^114^ Inability to account for maternal secretor status is a possible explanation for the discordant results reported for infant secretor status across studies, but this requires further investigation.

### Factors affecting the infant gut microenvironment

#### Oral polio vaccine interference

It has now been well demonstrated that concurrent administration of the first dose of oral polio vaccine (OPV) with the first dose of ORV decreases RV-IgA seroconversion and concentration.^115–117^ This phenomenon is likely due to interference from intestinal OPV replication, which is most robust following the first dose of OPV and may out-compete ORV replication. With planned withdrawal of OPV as a part of the global polio eradication effort, this may become less of a factor if more countries can transition to inactivated polio vaccines (IPV). Unfortunately, this remains a challenge due to higher cost and supply issues of IPV and recent resurgences in outbreaks due to circulating vaccine-derived polioviruses, particularly due to Sabin strain 2.^118–120^ Genetically stable novel OPV vaccines (nOPV) that are less likely to revert to neurovirulence than Sabin strains are currently under development. nOPV against type 2 poliovirus is furthest in development, but since nOPV2 shedding in infants was comparable to historically observed rates of monovalent OPV2 shedding shortly after vaccination,^121^ nOPVs seem unlikely to differ in inhibitory effect on ORVs, although this remains to be determined.

#### Coinfections and microbiome

In Bangladesh, infection with non-polio enteroviruses at the time of ORV administration was associated with decreased RV-IgA seroconversion and concentration and increased vaccine failure.^122^ However, similar studies conducted in India and Zimbabwe found opposite effects, with enterovirus quantity positively associated with ORV RV-IgA seroconversion, although quantity of non-polio enteroviruses in these studies were inferred rather than directly measured due to lack of methods that specifically detect non-polio enteroviruses but not Sabin-strain polioviruses.^115,117^ It is possible that timing of vaccination could have affected these findings, as the Bangladesh study provided the first Rotarix dose at 10 weeks, concurrent with the second OPV dose, while infants in the Zimbabwe and India studies received their first doses of Rotarix and OPV concurrently at 6 weeks. No consistent pathogen-specific effects, including enterovirus, at the time of vaccination on ORV response have thus been observed.

The role of infant intestinal microbiome is an area of active interest. Investigators have reported significant differences in infant gut microbiota in Rotarix RV-IgA seroresponders and non-responders in cohorts from Pakistan and Ghana.^123,124^ In Pakistan, vaccine response correlated with higher relative abundance of bacteria belonging to *Clostridium* cluster XI and Proteobacteria, while in Ghana vaccine responders had an increased abundance of *Streptococcus bovis* and decreased abundance of Bacteroidetes compared to non-responders. This study was limited by very low RV-IgA response (15% seroconversion). In both studies, infants were also compared to a cohort of Dutch infants, with responders sharing greater similarity in overall microbiome composition with healthy Dutch infants, who were presumed to have good ORV response, compared to non-responders. In contrast, no consistent differences in microbiota composition or alpha or beta diversity could be detected in Rotarix seroconverters vs non-converters in India, although slightly more pre-vaccination bacterial taxa were observed in those who shed vaccine after the first dose.^115^ In Dutch adults who underwent microbiota modification via narrow- vs- wide-spectrum oral antibiotics followed by a single dose of Rotarix, those who received narrow-spectrum antibiotics (oral vancomycin) had an increased rate of RV-IgA boosting, defined as a two-fold increase, and increased frequency of fecal vaccine shedding compared to controls, suggesting that alterations in microbiota could have detectable effects on ORV response.^125^ Since all volunteers were adult males who had quite robust baseline RV-IgA levels, it remains unclear how these findings would translate to infants in LMIC.

An underexplored topic is maternal breast milk microbiome. Breast milk microbiome in mothers of neonates with symptomatic rotavirus infections due to neonatal G10P[11] rotavirus in India clustered differently from those with asymptomatic neonatal infection or uninfected neonates, with increased Enterobacter/Klebsiella relative abundance in breast milk of mothers of symptomatic infants.^126^ Similar findings were observed for infant fecal microbiome. However, the relevance of these findings for ORV response also remain uncertain. The effects of infant and maternal microbiome on ORV responses will likely remain an area of intense interest, with further insights to come.

#### WASH

The impact of household-level water, sanitation, and hygiene (WASH) interventions on Rotarix response was evaluated in Zimbabwe in a cluster-randomized 2 × 2 factorial trial, which also assessed improved feeding interventions on child health outcomes. WASH interventions were associated with a modest increase in seroconversion among vaccinated infants in WASH groups versus non-WASH groups, with an absolute difference of 10.6% (95% CI 0.54–20.7).^127^ Fewer infants in the WASH group were seropositive pre-vaccination, meaning this difference could have resulted from reductions in early RV exposure in the WASH group rather than a direct effect on vaccine response. However, exclusion of baseline-seropositive infants demonstrated a consistent effect, with an absolute difference of 9.8% (95% CI, −6-20.2). These results are tempered by the low rates of seroconversion observed, which was 35.4% in WASH infants who received both doses of vaccine. In this setting, WASH was not associated with significant reductions in rotavirus prevalence.^128^ The ability of household-level WASH alone to improve ORV performance thus appears to be limited.

#### Probiotics and zinc supplementation

Zinc deficiency is common in children in LMIC and has long been recognized as important in the treatment of pediatric diarrhea, with recent evidence suggesting a specific role in protection from rotavirus diarrhea.^22^ Therefore, zinc supplementation has been proposed as a potential adjunct to aid in response to rotavirus vaccination. Similarly, probiotics have received interest as another possible intervention to induce a gut microbiota more favorable to oral rotavirus vaccine response. A study in India randomized infants into four groups: probiotic (10^10^ *Lactobacillus rhamnosus* GG) plus oral zinc (5 mg daily); probiotic only with zinc placebo; probiotic placebo with zinc only; or probiotic placebo and zinc placebo, all starting one week before initiating the Rotarix vaccine series until 6 weeks after the second dose.^129^ Neither zinc nor probiotic was associated with significantly increased RV-IgA seroconversion, although a suggestion of benefit was noted for probiotic, with a 7.5% increase in seroconversion in all infants who received probiotic compared to those who received none (97.5% CI −1.4–16.2).

#### Environmental enteric dysfunction (EED)

EED is a subclinical disorder of gut function and inflammation that affects impoverished populations in LMIC, presumably due to high enteropathogen exposure. It is associated with intestinal villus blunting, impaired barrier function, and malabsorption.^130^ EED has long been proposed as an important variable in oral vaccine underperformance, but compelling evidence has proven difficult to produce, with large trials yielding conflicting results.^131^ The greatest barrier to this field has been lack of validated surrogate markers for EED: the current gold standard for diagnosis remains histopathologic examination of intestinal biopsy specimens, and an objective scoring index has only recently been developed.^132^ Numerous stool, serum, and urine biomarkers capturing specific aspects of EED-associated pathology (e.g. intestinal inflammation, permeability, malabsorption, translocation, etc.), have been evaluated in multiple settings with few consistent findings.^133,134^ Work to identify reliable noninvasive biomarkers as surrogates for biopsy-proven EED is currently ongoing, and may hopefully refine approaches toward EED detection and risk stratification in at-risk populations.^135^

## Factors impacting measurements of vaccine efficacy

At least part of the lower estimates of vaccine efficacy observed in LMIC may have to do with mathematical phenomena due to limitations in standard methods for measuring vaccine efficacy. For example, using data from a Rotarix trial performed in Bangladesh, researchers determined that traditional efficacy measurements that failed to account for immunity due to natural infections in the placebo group during periods of prior exposure underestimated overall efficacy by 7.1%, with a 13.5% increase specifically in year 2 efficacy when only evaluating rotavirus-naïve children.^136^ They further developed a model to simulate variation in year 2 efficacy using data from other regions representing a spectrum of rotavirus incidence rates and vaccine efficacies. This model suggested that underestimation of year 2 vaccine efficacy was greatest in settings with calculated efficacy near 50%, which generally corresponds to efficacy estimates for many LMIC.

Similarly, lack of accounting for decreased natural susceptibility to rotavirus infections in individuals with non-secretor HBGA status was shown to decrease reported estimates of vaccine efficacy in Bangladesh.^107^ In this study, efficacy against rotavirus-associated AGE of any severity among non-secretors was 31.7%, compared to 56.2% among secretors. The reduced efficacy observed among non-secretors appeared to be due to the natural protection afforded by non-secretor status in unvaccinated infants (which approached a 50% risk reduction compared to unvaccinated secretors): decreased susceptibility in non-secretors meant that vaccination in this group offered little additional protection. The difference in rotavirus incidence in vaccinated versus unvaccinated non-secretors was thus minimal, resulting in a reduced effect size. This led to a mathematical reduction in efficacy that did not reflect the biological mechanism for this outcome. Similar effects may provide an incremental contribution to lower efficacy estimates in other trials, particularly in regions with a high population prevalence of non-secretors. In contrast, a study in Malawi found that non-secretors had a reduced risk of rotavirus vaccine failure, suggesting that secretor status does not impact vaccine performance estimates, but this analysis was performed using a case–control design and may not be directly comparable to efficacy estimates obtained from prospective clinical trials.^113^

Difficulties in attribution of diarrheal etiology have also come under increased scrutiny. Among infants in LMIC, coinfections with multiple enteropathogens are frequently observed.^3,137^ In the context of a vaccine trial, vaccinated infants with a rotavirus-positive diarrheal episode would be considered a case of vaccine failure. However, significant misattribution of diarrhea incidence to rotavirus could occur if these episodes were also frequently associated with coinfections with other etiologic agent of gastroenteritis, but for which testing was not performed. Post-vaccination, it is possible that rotavirus infections would be more likely to be asymptomatic, as is observed in subsequent episodes of natural infection,^14^ and therefore more frequently seen in the context of coinfection rather than symptomatic monoinfection. A high incidence of diarrhea due to other undetected pathogens could thus confound vaccine outcome measures in rotavirus efficacy trials. In Botswana, a recent study did not detect a difference in rotavirus vaccine effectiveness in patients with intestinal coinfections compared to those without.^138^ In this study, co-pathogens were detected using either in-house multiplex PCR (nine targets) or a commercial gastrointestinal PCR panel (15 targets). A significant limitation of this study was a small sample size, which may have underpowered the study to reach more definitive conclusions, particularly as there did appear to be a possible effect of coinfections detected using the in-house panel: vaccine effectiveness was 48% in the coinfection group and 62% in the group with rotavirus infection alone. Similarly, detection of an even broader range of coinfections using Taqman Array Card (TAC) was performed in the Rotavac vaccine efficacy trial in India.^139^ In this study, accounting for enteric coinfections led to an 11.3% increase in vaccine efficacy. Similar effects could help explain a portion of the lower vaccine efficacy estimates observed in LMIC, but further work will be required to resolve this issue.

Clearly, the underperformance of current ORVs in LMIC is multifactorial. A small but important component of the reduced efficacy observed in these regions may have to do with limitations in approaches for measuring vaccine performance. However, a spectrum of biological factors is also clearly involved, meaning any single intervention is unlikely to fully achieve levels of ORV performance comparable to high-income settings. Such interventions (e.g. delayed or extra dosing, booster doses, increased vaccine inoculum, staggered OPV administration, micronutrient or probiotic supplementation) have had limited success in improving oral vaccine responses, as detailed above. Even if they demonstrated larger effects, none are easily implemented as they would all require large-scale restructuring of current WHO vaccine schedules or add significant cost to the global rotavirus vaccine effort, at a time when access to current vaccines is already suboptimal. In 2019, an estimated 85 million children still did not have access to vaccine.^140^ Therefore, next-generation vaccines will likely be needed to fully bridge this gap. A number of newer vaccines are currently undergoing clinical evaluation or development, including oral, live-attenuated vaccines and parenteral non-replicating rotavirus vaccines.

## Newer and next-generation rotavirus vaccines

An overview of newer and next-generation vaccines is provided in Table 3.

**Table 3.** Overview of newer and next-generation vaccines under evaluation or in development

Vaccine type	Manufacturer/Developer	Composition	Status
**Oral, live-attenuated**			
RV3-BB	PT BioFarma	Monovalent G3P[6] (strain RV3) neonatal human rotavirus	Phase 2/3
**Non-replicating, parenteral**			
P2-VP8*	PATH SK Bioscience	Trivalent subunit vaccine: tetanus toxoid P2 fused to P[4] (strain DS1), P[6] (strain 1076), and P[8] (strain Wa) VP8*	Phase 3
CDC-9	CDC Serum Institute of India Pvt. Ltd Zhifei Lvzhu Biopharmaceutical Co., Ltd.	Monovalent inactivated human rotavirus strain CDC-9	Preclinical
116E	Bharat Biotech International Ltd.	Monovalent inactivated human rotavirus strain 116E	Preclinical
VLP VP2/6/7	Mitsubishi/Medicago	Virus-like particle	Phase 1
VLP VP2/4/6/7	Baylor College of Medicine	Virus-like particle	Preclinical
VP6 + norovirus	Tampere University, Finland	Nanoparticle: VP6 nanotubes or microspheres + norovirus viral-like protein admixture	Preclinical
VP6 + norovirus	Cincinnati Children’s Hospital Medical Center	Sub-viral particles (norovirus S or P) expressing rotavirus VP8* particle	Preclinical
VP8* mRNA	CureVac	VP8* mRNA	Preclinical

Newer formulations of already licensed vaccines are not included.

### Newer oral vaccines

#### RV3-BB

A promising newer ORV is RV3-BB (PT BioFarma, Bandung, Indonesia). This vaccine consists of a neonatal strain of G3P[6] human rotavirus (RV3). As a naturally attenuated neonatal strain, it was found to cause wild-type asymptomatic infection very early in life and is infectious and immunogenic in spite of high levels of maternally derived serum or breast milk antibodies, providing a rational alternative path for improving oral rotavirus vaccine responses.^141^ RV3-BB demonstrated efficacy in a double-blind, placebo-controlled Phase 2b trial in Indonesia using both infant and neonatal dosing.^142^ In this trial, participants were randomized to neonatal dosing at 0–5 days, 8 weeks, and 14 weeks of age, infant dosing at 8, 14, and 18 weeks of age, or placebo. Vaccine efficacy against severe rotavirus AGE through 18 months was 75% (95% CI, 44–91) in the neonatal-schedule group and 51% (95% CI, 7–76) in the infant-schedule group. Efficacy was even higher for the first year of life, estimated at 94% (95% CI, 56–99) in the neonatal-schedule group and 77% (95% CI, 31–92) in the infant-schedule group. A phase 2 dose-ranging study to confirm appropriate dosing in African infants has been performed in Malawi, although results are not yet available (ClinicalTrials.gov NCT03483116). A phase 3 trial in Indonesia using RV3-BB produced under Halal manufacture (PT BioFarma) is scheduled to commence in mid-2020; pending trial results, developers plan to submit for local licensure and national roll-out in Indonesia, followed by WHO pre-qualification (Julie Bines, personal communication).

Other unique characteristics of RV3-BB make it an intriguing addition to the ORV repertoire. For example, high rates of vaccine take were observed irrespective of infant HBGA status, and immune response appeared to be less susceptible to interference from OPV, with vaccinated infants who received concurrent OPV demonstrating similar rates of antibody seroconversion and GMT compared to vaccinated infants who received IPV.^143,144^ Interestingly, Rotavac is also derived from a neonatal rotavirus strain (G9P[11]) but showed efficacy levels similar to those of other ORVs in LMIC. Whether strain-specific differences or adoption of a neonatal dosing regimen means RV3-BB’s promising findings from Indonesia can be replicated in other locations remains to be seen.

### Non-replicating rotavirus vaccines (NRRVs)

Given the ongoing challenges with oral vaccine underperformance, the potential of parenteral NRRVs has received intense interest. The potential advantages of such vaccines include: circumventing the so-called “tropical barrier” presented by multiple factors (e.g. EED) that prevent successful take of live-attenuated vaccines in the gut, hopefully leading to improved efficacy; reduced cold-chain footprint and cost; opportunity via sequential scheduling strategies (e.g., “prime-boost”) to augment (rather than replace) current ORV programs; and development of combination vaccines to facilitate administration and improve access. A meeting of NRRV vaccine developers was organized by PATH and held in Geneva, Switzerland in June 2019; additional information regarding the following vaccine candidates are available in the meeting proceedings^145^. An overview of NRRV candidates currently in development is provided in Table 3.

#### Subunit vaccines

Furthest along in development is a trivalent P2-VP8* subunit vaccine developed by PATH. This vaccine consists of a truncated segment of VP8* that contains all known VP8*-specific neutralizing epitopes fused to the P2 tetanus toxoid T cell epitope.^146^ An initial monovalent formulation consisting of VP8* derived from G1P[8] Wa strain rotavirus was well tolerated and immunogenic in a phase 1 trial among South African infants.^147^ All children received Rotarix following completion of study vaccination, which provided a unique opportunity to assess mucosal immunity: if recipients of study vaccine inhibited shedding of Rotarix (a homotypic P[8] virus) following oral challenge, this would suggest that the antibodies induced by the parenteral vaccine provided sterilizing immunity in the gut mucosa. Indeed, those who received study vaccine had reduced frequencies of stool Rotarix shedding following the first Rotarix dose compared to placebo recipients, with the overall percent reduction in shedding at any measured time point in vaccinated infants compared to placebo recipients ranging from 49% to 66%, depending on the investigational vaccine dose received. However, serum neutralizing responses to heterotypic rotavirus strains were poor overall. Based on these results, a trivalent formulation that added DS1 strain P[4] VP8* and 1076 strain P[6] VP8* underwent a subsequent phase 1/2 trial in the same location. In this study, the trivalent formulation was well tolerated and robust IgG and neutralizing seroresponses to P[4], P[6], and P[8] rotaviruses were observed, although serum IgA responses to each individual antigen were modest (20–34%) across all three dosages evaluated.^148^ Similar to the previous trial, Rotarix was given to all infants after study vaccination, and post-Rotarix shedding data were collected in a subset of infants: compared to placebo, significant reductions in shedding were again observed in infants who received parenteral vaccine, but only at the highest dose (90 µg) administered, which is quite high for a typical infant vaccine injection.^148^

Since functional antibodies in the gut are primarily IgA, it remains to be seen whether strong serum IgG and neutralizing antibodies induced by this vaccine can mediate sufficient mucosal immunity to prevent symptomatic AGE due to rotavirus. Observational data demonstrating the importance of maternally derived serum RV-IgG in infants (see above) gives reason for optimism, but the extent to which VP8*-specific antibodies alone can mediate this effect is unknown. A commercial partner, SK Bioscience (Seoul, South Korea) has been identified for this vaccine and a multinational phase 3 efficacy trial evaluating three 90ug doses of trivalent P2-VP8* at sites in Africa (Ghana, Malawi, Zambia) and India is currently in progress (ClinicalTrials.gov NCT04010488).

#### Inactivated vaccines

A monovalent, heat-inactivated whole virus vaccine consisting of G1P[8] strain CDC-9 human rotavirus developed by the Centers for Disease Control and Prevention is currently in development. Unlike other rotavirus strains, CDC-9 is reported to grow to high titer and demonstrate very high stability in the infectious, triple-layered particle form.^149,150^ Since the mechanisms of immunity and the effectors necessary or sufficient for protection from rotavirus AGE remain incompletely understood, an inactivated, intact virion is an attractive choice for a non-replicating vaccine, since this would promote presentation of all surface neutralizing epitopes. This vaccine has undergone extensive pre-clinical evaluation, demonstrating strong induction of homotypic and heterotypic serum neutralizing antibody responses and reductions in fecal virus shedding upon oral challenge in vaccinated animals compared to placebo in multiple animal models,^150–153^ including when delivered intradermally using a novel-coated skin microneedle patch.^152,154^ Two commercial partners, Serum Institute of India Pvt. Ltd. (Pune, India) and Zhifei Lvzhu Biopharmaceutical Co., Ltd. (Beijing, China) have reported initiation of Good Manufacturing Process (GMP) production and regulatory approvals processes in preparation for phase 1 clinical trials^146^.

Bharat Biotech is reportedly also developing an inactivated version of Rotavac for parenteral administration. No data regarding its progress or performance are publicly available to date^146^.

#### Virus-like particle (VLP) and nanoparticle vaccines

Double- or triple-layered VLPs containing various combinations of all major capsid layer proteins (VP2, VP6, VP7, and/or VP4) expressed in recombinant baculovirus-infected insect cell culture have been developed as potential NRRV candidates by Baylor College of Medicine (Houston, TX).^153,155^ These candidates have also undergone extensive preclinical testing in multiple animal models, including the demonstration of broad heterotypic neutralizing antibody induction in mice immunized with a G1 VP7-containing construct.^156–158^ Immunization with this approach is now used to stimulate generation of protective antibodies in a commercial bovine colostrum formulation that was approved by the United States Department of Agriculture in 2017 for passive vaccination to prevent calf scours (First Defense Tri-Shield, Immucell, Portland, ME).^159^ Despite successful use in veterinary medicine and the growing list of successful human VLP-based vaccines, insect cell-produced VLP vaccine candidates for rotavirus have yet to advance past the preclinical phase.

More recently, Medicago Inc. (Quebec City, Canada), a subsidiary of Mitsubishi Tanabe Pharma Corporation (Osaka, Japan), has applied its proprietary technology using plant-based VLP production toward development of a rotavirus VLP vaccine. A randomized, placebo-controlled, descending age dose-escalation phase 1 trial to evaluate the safety and immunogenicity of their candidate vaccine MT-5625 in adults, toddlers, and infants has been completed in Australia and South Africa (ClinicalTrials.gov NCT03507738), but results have not yet been released.

Several nanoparticle-based vaccines are also in the pre-clinical phase of development. A group at Tampere University, Finland, has developed a combined rotavirus/norovirus vaccine candidate containing self-assembled rotavirus VP6 nanoparticles expressed in recombinant baculovirus-infected insect cells, including in both nanotube and microsphere configurations.^160,161^ Interestingly, VP6 appeared to have the additional benefit of acting as an adjuvant for norovirus immunogenicity in mice.^161^ Another proposed combination rotavirus/norovirus construct under development at Cincinnati Children’s Hospital Medical Center (Cincinnati, OH) uses rotavirus VP8* expressed on the surface of recombinant norovirus P or S subviral particles. Noroviruses contain a single outer capsid structural protein, VP1, which contains two domains, the shell (S) and protrusion (P) domains, each of which can be independently expressed to form self-assembling sub-viral particles as a platform for foreign antigen presentation.^162^ Both P and S formulations were immunogenic in mice and the S-particle construct reduced fecal viral shedding in vaccinated mice following oral challenge with a VP8*-homologous strain.^162,163^

#### mRNA vaccines

Finally, the Bill and Melinda Gates Foundation has partnered with CureVac (Tübingen, Germany) to leverage their mRNA-based technologies toward development of a rotavirus VP8* mRNA vaccine^146^. This project is also in the preclinical stage of development and public data are not yet available. The overall mRNA approach has been evaluated as a plausible approach for vaccine development.^164^

#### Future vaccine prospects

The recent development of a plasmid-based reverse genetics system for rotavirus is an important breakthrough with significant potential to accelerate rotavirus research. Hopefully, this new technology will augment existing platforms (as detailed above) and assist in development of new vaccine candidates.^165^

## Additional challenges

In addition to the issues related to vaccine development, clinical evaluation in the field remains challenging. Next-generation vaccines will likely be targeted specifically to LMIC, where large-scale field trials remain more difficult to perform, often due to relative limitations in research infrastructure, supplies procurement, and transportation, posing added challenges to overall research capacity. New studies may need to carefully consider and account for the growing list of biological and mathematical phenomena that may confound accurate measures of true vaccine efficacy. Furthermore, the availability and use of multiple WHO pre-qualified ORVs means that placebo-controlled efficacy trials are now difficult to justify ethically. Future studies may need to rely on active comparator arms, as was the approach adopted for the current phase 3 trivalent P2-VP8* efficacy study (NCT04010448).

Under such conditions, one potential approach would be using bridging studies comparing immunogenicity outcomes. The currently accepted standard for ORV immunogenicity, RV-IgA, may be a plausible approach for new oral vaccines, despite being a sub-optimal correlate of protection in LMIC.^69,166^ RV-IgA is likely a non-mechanistic correlate of protection, as immunized infants without a post-immunization RV-IgA response still appear to have greater protection from rotavirus diarrhea than non-immunized infants, and similarly those with high RV-IgA may still develop disease.^69,166^ Nevertheless, current evidence suggests that it may be “reasonably likely to predict clinical benefit,” although it has not been validated as a true surrogate endpoint.^67,166^ However, RV-IgA may not be as applicable for assessment of NRRVs, as immune responses induced by parenteral vaccination may differ from those induced by live-attenuated oral vaccines. Until a true rotavirus immune correlate of protection that can be induced by both parenteral and oral vaccines can be confirmed, trials of NRRVs are likely to ultimately require clinical endpoints. Non-inferiority studies may be a reasonable option, but opportunities to demonstrate improved efficacy compared to current vaccines may be significantly hindered by the substantially larger sample sizes required.

### Conclusions

Current ORVs have had substantial impact on rotavirus morbidity and mortality throughout LMIC, but continue to underperform in these settings relative to high-income countries. Coupled with ongoing challenges related to ORV cost, supply, and access, the full global potential of rotavirus vaccines is still not being realized. Overcoming this challenge will require a multifaceted approach, taking into consideration the multiple factors that impact ORV performance and the likely need for newer and next-generation vaccines. Ongoing exploration to identify better immune correlates of protection following vaccination for both ORVs and NRRVs are needed. Indeed, the landscape for rotavirus vaccines may substantially change in coming years, and hopefully the second decade following universal WHO rotavirus vaccine pre-qualification will see increasing progress toward reducing rotavirus vaccine underperformance and rotavirus-associated morbidity and mortality among children in LMIC.
